# Targeting L1 cell adhesion molecule expression using liposome-encapsulated siRNA suppresses prostate cancer bone metastasis and growth

**DOI:** 10.18632/oncotarget.2478

**Published:** 2014-09-16

**Authors:** Shian-Ying Sung, I-Hui Wu, Pei-Hsin Chuang, John A. Petros, Hsi-Chin Wu, Hong-Jie Zeng, Wei-Chien Huang, Leland W. K. Chung, Chia-Ling Hsieh

**Affiliations:** ^1^ The Ph.D. Program for Translational Medicine, College of Medical Science and Technology, Taipei Medical University, Taipei, Taiwan; ^2^ Graduate Institute of Cancer Biology, China Medical University, Taichung, Taiwan; ^3^ Department of Urology, Emory University, Atlanta, GA, USA; ^4^ School of Medicine, China Medical University, Taichung, Taiwan; ^5^ Department of Urology, Atlanta VA Medical Center, Decatur GA, USA; ^6^ Department of Medicine, Cedars Sinai Medical Center, Los Angeles, CA, USA; ^7^ Department of Biotechnology, Asia University, Wufeng, Taichung, Taiwan

**Keywords:** L1 cell adhesion molecule (L1CAM), prostate cancer, bone metastasis, gene therapy, small interfering RNA (siRNA)

## Abstract

The L1 cell adhesion molecule (L1CAM) has been implicated in tumor progression of many types of cancers, but its role in prostate cancer and its application in targeted gene therapy have not been investigated. Herein, we demonstrated that the L1CAM was expressed in androgen-insensitive and highly metastatic human prostate cancer cell lines. The correlation between L1CAM expression and prostate cancer metastasis was also validated in serum samples of prostate cancer patients. Knockdown of L1CAM expression in prostate cancer cells by RNA interference significantly decreased their aggressive behaviors, including colony formation, migration and invasion *in vitro*, and tumor formation in a metastatic murine model. These anti-malignant phenotypes of L1CAM-knockdown cancer cells were accompanied by G0/G1 cell cycle arrest and suppression of matrix metalloproteinase (MMP)-2 and MMP-9 expression and nuclear factor NF-κB activation. *In vivo* targeting of L1CAM expression using liposome-encapsulated L1CAM siRNAs effectively inhibited prostate cancer growth in mouse bone, which was associated with decreased L1CAM expression and cell proliferation by tumor cells. These results provide the first evidence for L1CAM being a major contributor to prostate cancer metastasis and translational application of siRNA-based L1CAM-targeted therapy.

## INTRODUCTION

Prostate cancer is the most frequently diagnosed cancer in men and the second leading cause of cancer deaths among men in the United States [1]. The most common site of prostate cancer metastasis is in bone, with skeletal metastases identified at autopsy in up to 90% of patient dying from the disease [2]. Skeletal metastasis results in significant complications including severe pain, pathological fractures and spinal cord compression, bone-metastasis-evoked cranial neuropathy from base-of-skull syndromes, anemia, and infection [3]. While localized prostate cancer can be cured, patients with bone metastasis and resulting complications often have a poor prognosis and a median survival of 1~3 years [4]. Currently, androgen deprivation therapy is the first-line therapy for metastatic prostate cancer. However, the disease often progresses to the androgen-independent bone-metastatic stage. The only main treatment options then left are chemotherapy and radiotherapy, which cause unpleasant side effects and generally fail to induce long-term remission. To the present, despite the development of bone-targeted strategies, bone metastases are still considered incurable [5]. Novel therapeutic approaches based on a mechanistic understanding of prostate cancer metastasis and survival in the bone are needed to further improve the prospects for survival of men with hormone-refractory prostate cancer metastasis.

It has become clear that adhesion molecules and adhesion processes are essentially involved, although to variable levels, in all steps of the metastatic cascade [6]. The L1 cell adhesion molecule (L1CAM) belongs to the immunoglobulin superfamily, which is characterized by an extracellular region of multiple immunoglobulin-like domains and fibronectin type III repeats followed by a highly conserved cytoplasmic domain [7]. In addition to cell-surface localization, the extracellular domain of L1CAM can be released from the cell surface via proteolytic cleavage involving proteinases, such as plasmin and ADAM10 [8, 9], thus allowing deposition of the L1CAM in the extracellular matrix (ECM). The L1CAM was first described as a neural CAM based upon a restricted distribution [10], where it is involved in the control of cell migration, neurite extension, and prevention of cell death [11]. The L1CAM was recently identified as a key mediator of tumor progression due to its upregulation in a variety of human tumors, including melanomas [12], renal cancer [13], lung cancer [14], mesotheliomas [15] oral cancer [16], ovarian carcinoma [17], and hepatocellular carcinoma [18] with high correlations with cancer progression. In some malignancies, the level of the L1CAM is also a significant indicator of subsequent metastasis and reduced patient survival [12, 19, 20]. These observations further support the L1CAM being a potential therapeutic target in metastasis. To date, there is limited literature on the link between the L1CAM and prostate cancer. Calvo et al. [21] reported changes in L1CAM gene expression during prostate cancer progression in a transgenic mouse model. However, the biological function of the L1CAM in human prostate cancer has not yet been determined and remains to be excluded.

RNA interference (RNAi) is a natural endogenous mechanism for silencing gene expression [22, 23] that recently has provided new opportunities for gene therapy through the specific extinction of targeted gene(s) in human diseases [24]. Unlike traditional pharmacological inhibitors, RNAi-based therapeutics can inhibit all classes of gene targets, including extracellular and intracellular targets and mutant alleles, regardless of the function of the gene product with high selectivity and potency [25], therefore presenting an invaluable tool for personalized cancer therapy. Major targets for RNAi cancer therapy include oncogenes and genes that are involved in angiogenesis, metastasis, survival, antiapoptosis, and resistance to chemotherapy. As the L1CAM recently emerged as a key driver in cancer cell growth and metastasis, it might serve as a potential RNAi target in advanced cancers. In the present study, we focused on defining the role and specific mechanisms of L1CAM's involvement in the disease progression of prostate cancer and investigating the feasibility and efficacy of using an RNAi approach for the *in vivo* targeting of L1CAM expression for treating human prostate cancer bone metastasis.

## RESULTS

### L1CAM expression is correlated with the metastatic potential of human prostate cancer cells

**Figure 1:**
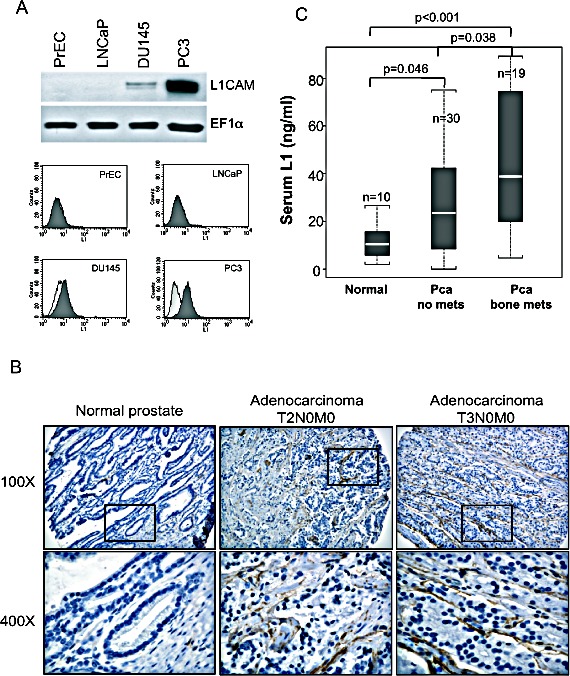
Detection of L1 cell adhesion molecule (L1CAM) expression in prostate cancer cell lines and clinical specimens (A) Representative Western blotting (top) and flow cytometric (bottom) analyses of L1CAM expression in LNCaP, DU145, and PC3 human prostate cancer cell lines and PrEC normal prostate epithelial cells. EF1-α protein levels are shown for various loading quantities of cell lysates. Cell lines stained with saturated amounts of monoclonal antibodies recognizing the L1CAM (shaded histogram) and isotype control antibody (unshaded histogram) were evaluated by a FACS analysis. (B) Human prostate tissue arrays were subjected to immunohistochemical analyses of L1CAM expression. Representative images from tissues with different pathologic characteristics at a magnification of 100x and enlargement (400x) of the area in the box are shown. (C) Serum L1CAM (L1) levels in a normal population (Normal) and prostate cancer patients with prostate-confined tumors (Pca no mets) and with bone metastases (Pca bone mets) were detected by an ELISA, n, sample number. Distributions of serum L1 across groups are shown as box plots. Significant differences were analyzed by the Wilcoxon rank sum test.

To examine whether the L1CAM is associated with prostate cancer progression, we first analyzed L1CAM expression in normal and several available prostate cancer cell lines by Western blotting and a flow cytometric analysis. L1CAM expression (Fig. 1A) was highly detected in the cell lysate and on the cell surface of androgen-independent and bone metastatic PC3 cells. DU145 cells derived from metastatic lesions in the dura mater expressed lower levels of the L1CAM compared to PC3 cells, whereas androgen-dependent LNCaP with low metastatic potential and normal prostatic epithelial PrEC cells exhibited no L1CAM expression. We further investigated L1CAM expression in a prostate adenocarcinoma tissue microarray by IHC. No positive staining was observed in normal prostatic glands in any (16 cores) normal prostate tissues. Staining of the L1CAM was occasionally detected in 8% (6 of 72 cores) of tumor tissues, which were classified as carcinoma in situ with no regional lymph node or distant metastasis (T2N0M0 and T3N0M0), with major localization at the interphase between the tumor and stroma (Fig. 1B).

Considering that DU145 and PC3 cell lines are derived from prostate cancer metastases at distant sites and express the L1CAM, we next examined whether L1CAM expression was associated with the status of prostate cancer distant metastasis. Prostate cancer cells preferentially metastasize to bone. Tissue resources of prostate cancer bone metastases are rare and difficult to collect. The ectodomain of the L1CAM can be shed and detected in serum samples of ovarian and uterine cancer patients [19, 26]. Alternatively, we examined whether L1CAM expression was correlated with the cancer metastasis status using sera from normal populations and prostate cancer patients with localized tumors or bone metastases. An ELISA analysis of L1CAM levels in conditioned media from PC3 and DU145 cells (296.1±0.67 and 29.0±1.34 ng/ml, respectively) confirmed that the ectodomain was shed by metastatic prostate cancer cells. In clinical specimens (Fig. 1C), mean serum L1CAM levels in bone-metastatic prostate cancer patients (45.0±27.2 ng/ml, n=19) were significantly higher than those in patients with prostate-confined tumors (28.4±22.2 ng/ml, n=30, p<0.05) and normal controls (12.1±8.6 ng/ml, n=10, p<0.001). Although patients with only localized prostate cancer had higher levels of serum L1CAM than normal populations, there was no correlation with the Gleason staging (data not shown). These results suggest that the major function of the L1CAM in prostate cancer progression is in the late stage of cancer metastasis rather than during primary tumor growth.

### Downregulation of the L1CAM by siRNA inhibits prostate cancer cell metastasis *in vivo*

**Figure 2:**
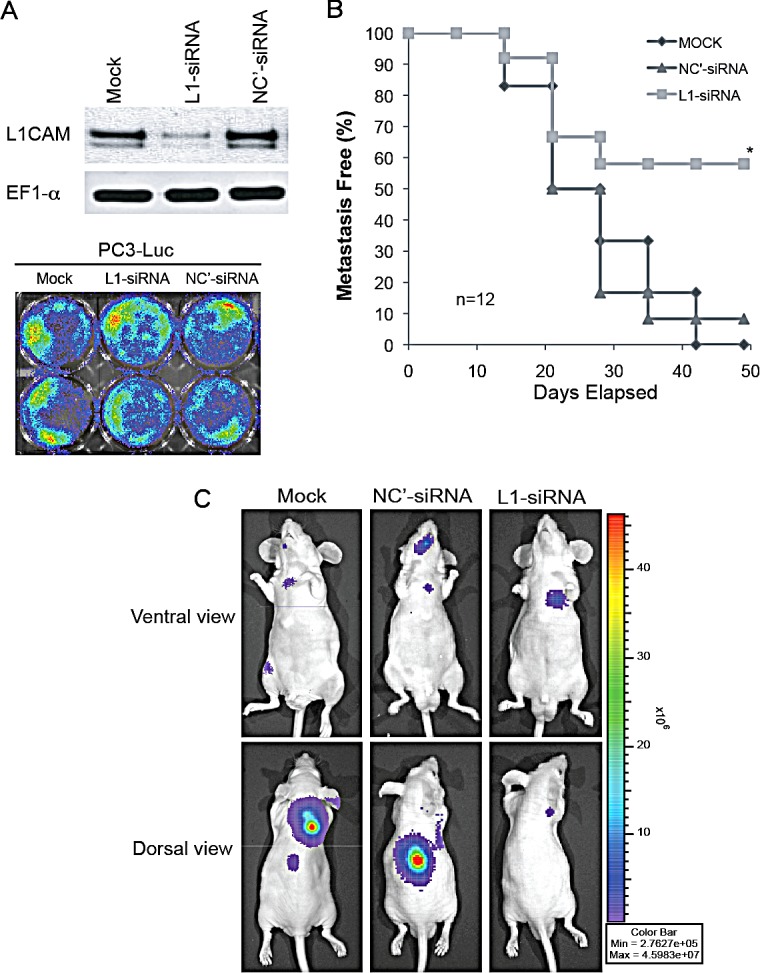
Effects of L1 cell adhesion molecule (L1CAM) downregulation on tumor metastasis in a PC3 xenograft model (A) L1CAM and luciferases expressions in PC3-Luc cells that had been transfected with L1CAM-siRNA (L1-siRNA), EFGP negative control (NC')-siRNA, or vesicle alone (mock) were compared by a Western blot analysis (top) and bioluminescent imaging (BLI; bottom), respectively. (B, C, D) PC3-Luc cells with the indicated transfection were injected into the left ventricle of nude mice. Mice developing visceral metastases were evaluated by BLI weekly. (B) A Kaplan-Meier analysis of the percentage of metastasis-free mice over time; n, mouse number per group; * p≤0.001 vs. the mock control. (C). Ventral and dorsal views of bioluminescent images of a representative mouse in each group taken at week 7 after cell injection. Signals were adjusted to the same color scale for each image.

To assess whether L1CAM can be a novel RNAi target for prevention or treatment of disseminated prostate cancer, we knocked down L1CAM expression in highly metastatic PC3 cells using siRNA. The metastatic potential of luciferase-tagged PC3 (PC3-Luc) cells transfected with siRNA against the L1CAM gene or the EFGP as a negative control, or mock-transfected cells was examined *in vivo* by injecting cells into the left ventricle of nude mice. This intracardiac model recapitulates the late steps in cancer metastasis, specifically tumor cell dissemination, survival, invasion, colonization, and distant growth [27]. We confirmed the L1CAM gene knockdown efficacy by L1CAM siRNA and equal bioactivity of the luciferase reporter among PC3-Luc transfectants using quantitative BLI prior to injection into animals (Fig. 2A). While mice receiving mock- and control siRNA-transfected PC3-Luc cells developed visually evident BLI metastases in as high as 100% (12/12) and 90% (11/12) of the populations, respectively, metastatic tumor growth of L1CAM siRNA-transfected PC3-Luc cells was only detected in fewer than 50% (5/12) of the mice at 7 weeks after the injection (Fig. 2B). In addition, although tumor-bearing mice revealed no significant differences in the distribution of metastases to specific areas, such as the neck back, chest, hind limb, and craniofacial regions among the three transfected groups (Table 1), the whole-body bioluminescent intensity of mice carrying L1CAM siRNA-transfected PC3-Luc tumors was 1 order of magnitude less than those of mock- and negative control shRNA groups (Fig. 2C). These results demonstrated that downregulation of the L1CAM by sequence-specific siRNA reduced the metastatic potential of prostate cancer cells to colonize a second organ.

**Table 1:** Incidences of metastases at different sites in tumor-bearing mice 50 days after an intracardiac injection

	Site of metastasis				
Transfectant	mandible	hind limb	spine/rib	lung	other sites
Mock	2/12 (16.7%)	2/12 (16.7%)	5/12 (41.7%)	5/12 (41.7%)	7/12 (58.3%)
NC'-siRNA	2/11 (18.2%)	2/11 (18.2%)	7/11 (63.6%)	3/11 (27.3%)	8/11 (72.7%)
L1-siRNA	0/5 (0%)	1/5 (20%)	3/5 (60%)	2/5 (40%)	3/5 (60%)

### Knockdown of the L1CAM decreases cell migration and invasion and suppresses MMP expression and NF-κB signaling in PC3 cells

**Figure 3:**
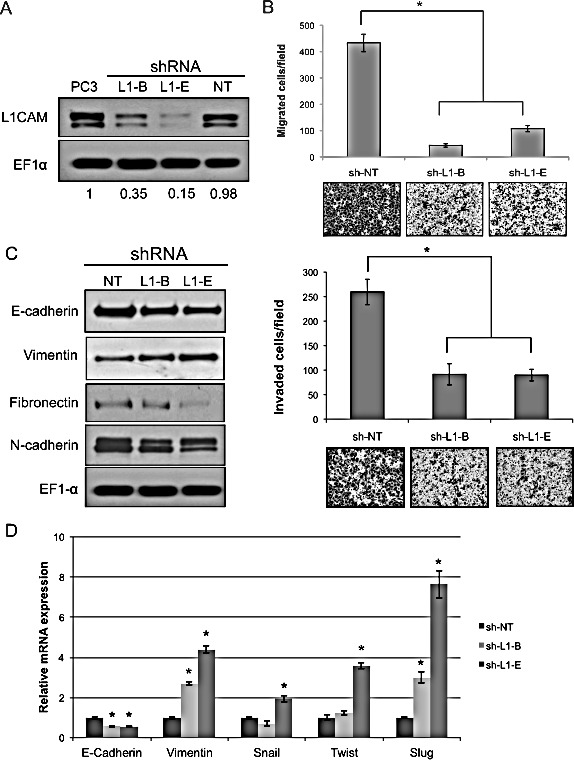
Effects of L1 cell adhesion molecule (L1CAM) gene knockdown on the migratory and invasive abilities of PC3 prostate cancer cells (A) Western blot analysis of L1CAM expression in PC3 cells stably expressing shRNA constructs targeting L1CAM (L1-B and L1-E) or a non-target control (NT). EF1-α protein levels are shown for various loading quantities. Changes in L1CAM protein expression in shRNA transfectants compared to parental PC3 after being normalized to the loading control are shown at the bottom. (B) Migration and invasion assays of L1CAM-shRNA-expressing PC3 cells. Cells that had migrated through the membrane (migration) or Matrigel (invasion) were stained with crystal violet 16 h and counted after cell plating. Assays were performed in three independent experiments in triplicates. Data are presented as the mean±SD of one representative experiment, and representative images (200x) of each line are shown at the bottom; * p≤0.05. (C) Western blot analysis of epithelial-mesenchymal transition (EMT) markers in L1CAM-shRNA-expressing PC3 cells. EF1-α was used as a loading control. (D) Real-time RT-PCR analysis for EMT markers. mRNA levels of the indicated genes in L1CAM shRNA-expressing PC3 cells are displayed as the fold change relative to non-targeting control cells. Data are representative of three independent experiments and shown as the mean ± SD. *p <0.05, **p <0.0001 versus sh-NT.

To understand the mechanisms underlying the antimetastatic action of L1CAM gene targeting of prostate cancer, we stably downregulated L1CAM expression in PC3 cells using lentiviral vector-delivered shRNA and examined the behavior of cells at different steps of the metastatic cascade *in vitro*. The attenuating effects of two distinct shRNA constructs (sh-L1-B and sh-L1-E) against the L1CAM were 65% and 85% when respectively compared to non-targeting control transfectant (sh-NT) and parental cells (Fig. 3A).

In the first instance, we evaluated the effect of L1CAM shRNA on the migratory and invasive abilities of PC3 cells by Boyden chamber assays. As shown in Figure 3B, the motility of PC3 cells that expressed L1CAM shRNA was significantly reduced by >70% compared to the non-targeting sh-NT control (p<0.001). The impaired migration of L1CAM shRNA-expressing PC3 cells was also observed in the condition of either low concentration or short time duration (Fig. S1), that is, below doubling time of the cells (Fig. S2A), thereby ruling out any effects of cell aggregation and cell proliferation on their migratory characteristics. Consistent with findings in the migration assay, approximately 65% inhibition of Matrigel invasion was observed in PC3 cells expressing either sh-L1-B or sh-L1-E, indicating that these suppressive effects on cell migration and invasion by L1CAM shRNA were probably not caused by an off-target event. Similar results were obtained in DU145 prostate cancer cell line that normally express L1CAM (Fig. S3), supporting the critical role of L1CAM in migratory and invasive property of prostate cancer.

The epithelial-mesenchymal transition (EMT) is the conversion of an epithelial cell into a mesenchymal cell, which increases cell motility [28]. To determine whether the reduction of prostate cancer invasiveness by L1CAM shRNA is through a reversal of the EMT, we evaluated expressions of genes associated with EMT phenotypes at the protein level. We found that while knockdown of the L1CAM caused decreased expressions of the mesenchymal markers, fibronectin and N-cadherin, the widely used EMT marker, vimentin, was enhanced rather than suppressed (Fig. 3C). On the other hand, the epithelial-specific surface molecule, E-cadherin, was downregulated in L1CAM-shRNA-expressing PC3 cells. Consistent with this protein profile, quantitative RT-PCR analysis (Fig. 3D) showed a 50~60% reduction in E-cadherin and a 2~4 fold increase in vimentin in L1CAM shRNA-expressing PC3 cells when compared with sh-NT control cells. Moreover, known transcription factors that repress E-cadherin expression, such as Slug, Twist and Snail [29], were conversely increased in L1CAM-deficient cells, further confirming that the altered gene expression caused by L1CAM knockdown was EMT-related. Levels of increased vimentin and decreased E-cadherin were correlated with the L1CAM knockdown effectiveness of shRNA, revealing an undescribed mechanism of the L1CAM on prostate cancer metastasis that contradicts its known function of promoting the EMT seen in other cancer types.

**Figure 4:**
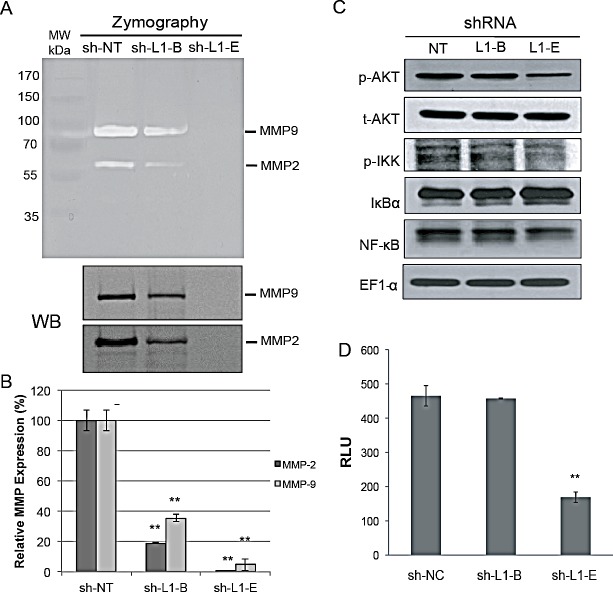
Effect of L1 cell adhesion molecule (L1CAM) shRNA on matrix metalloproteinase and nuclear factor NF-κB activation in prostate cancer PC3 cells (A) Gelatin zymographic (top) and Western blot (WB, bottom) analyses of MMP-2 and MMP-9 expression in conditioned medium (CM) from the shRNA-expressing PC3 cell lines. The positions of active MMP-2 and MMP-9 are indicated. (B) Quantitative real time RT-PCR analysis of transcriptional levels of MMP-2 and MMP-9. The relative MMP expression was normalized to the HSPCB housekeeping gene and plotted relative to the sh-NT control. Data are presented as the mean±SD of three independent determinations. ** p<0.001, compared to the sh-NT control group. (C) Western blot analysis of the Akt and NF-κB pathway. Total cell lysates were immunoblotted using antibodies against either the phosphorylated (p-) or total (t-) amount of the indicated proteins. (D) Luciferase reporter assay. Indicated shRNA-expressing PC3 cells were cotransfected with an NF-κB luciferase reporter construct and pCMV-β-galactosidase at a ratio of 5:1. After 48 h, luciferase activity was determined, normalized to β-gal activity, and is shown as relative luciferase units (RLU). Results are presented as the mean±SD of three independent determinations. ** p<0.001, compared to the sh-NT control group.

To elucidate whether L1CAM shRNA-mediated suppression of invasive phenotypes was associated with changes in MMPs, the expression and enzymatic activity of MMP-2 and MMP-9, which is associated with clinical disease progression to bone metastasis in prostate cancer patients [30], were determined in genetically modified PC3 cells. Data from both a Western blot analysis and zymography demonstrated that MMP-2 and MMP-9 were significantly decreased in L1CAM shRNA-expressing PC3 cells compared to vector control cells, with a stronger reduction in clone sh-L1-E than in clone sh-L1-B (Fig. 4A). The quantitative RT-PCR confirmed that the influence of MMPs expression by L1CAM shRNA is regulated by gene transcription (Fig. 4B). As the transcriptional factor, NF-κB, is important in MMP gene regulation [31], we further explored the relationship between L1CAM gene knockdown and the NF-κB pathway using a Western blot analysis. We found that there was decreased phospho-AKT and phospho-IKK and increased IκBα concomitant with downregulated NF-κB p65 expression levels in L1CAM shRNA-expressing PC3 cells (Fig. 4C), demonstrating that the AKT/IKK/NF-κB pathway was suppressed. In addition, treatment of PC3 cells with IKK inhibitor or NF-kB inhibitor (pyrrolidine dithiocarbamate; PDTC) attenuated their highly invasive phenotype (Fig. S4), providing a mechanistic evidence for the role of IKK/NF-κb signaling pathway in metastatic potential of prostate cancer cells. A pNF-κB-Luc reporter assay further confirmed inactivation of NF-κB's nuclear function upon L1CAM shRNA expression (Fig. 4D).

### Knockdown of the L1CAM inhibits cell-cell contact growth of PC3 cells by inducing G0/G1 cell-cycle arrest

**Figure 5:**
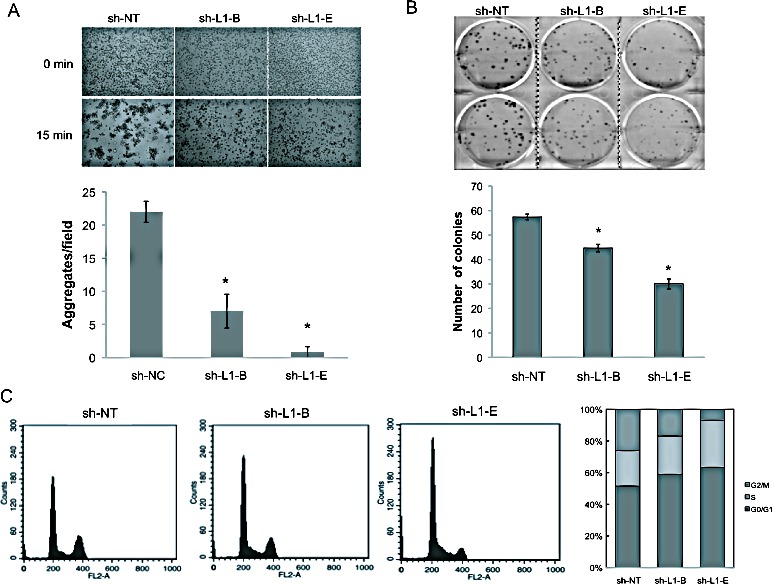
Effects of L1 cell adhesion molecule (L1CAM) knockdown on prostate cancer PC3 cell homophilic adhesions and growth (A) Cell aggregation assays of shRNA-expressing PC3 cells. Single cells were rotated for 15 min on a gyratory shaker, and the resultant cell aggregates were photographed (100x) under a phase-contrast microscope. Clusters consisting of more than 10 cells were counted, and results are expressed as the mean±SD of three independent determinations. * p<0.001, compared to the sh-NT control group. A representative photograph is shown at the top. (B) Colony formation assay. Indicated cells were seeded in six-well plates at a density of 100 cells per well, and cultured for 10 days. Then, cells were stained with crystal violet, photographed, and counted. Data are presented as the mean±SD of three independent experiments with duplicate wells per condition. * p<0.05, compared to the sh-NT control group. (C) FACS analysis of cell cycle distribution. Indicated cells were cultured for 16 h and then stained with propidium iodide for DNA content, and the acquired data were processed with the ModFit LT program. Results shown are a representative FACS profile of three independent experiments and a stacked column graph of the percentage of cells in each phase.

Previous studies showed that tumor cells tend to form cell clusters or spheroids in the circulation, thereby effecting suppression of anoikis and facilitating secondary tumor formation in distant organs [32]. To test whether L1CAM shRNA-mediated inhibition of prostate cancer metastasis is a critical mediator of disseminating prostate cancer cells survival in circulation, we first compared the ability of PC3 cells with or without L1CAM shRNA expression to form cell aggregates in single-cell suspensions. We found that non-targeting shRNA-expressing PC3 cells rapidly adhered to each other and generated large cell clumps in 15 min of incubation time (Fig. 5A). In contrast, shRNA-mediated downregulation of the L1CAM effectively decreased the homotypic cell-cell adhesion capacity of PC3 cells, leading to a dramatic reduction in the number and size of aggregates formed. In the colony-formation assay, upon low-density seeding, colony numbers of sh-L1-B- and sh-L1-E-expressing PC3 cells were 30±4.6 and 44±2.2 with respective inhibition ratios of 23% and 48% (p<0.01) compared to control shRNA-expressing cells (Fig. 5B), which further reveals a function of the L1CAM in supporting cell contact-dependent growth of tumor cells.

To characterize the antiproliferative effect exerted by L1CAM knockdown in more detail, we analyzed the cell-cycle distribution of shRNA-expressing cells using a flow cytometric analysis. As shown in Figure 5C, L1CAM shRNA-expressing PC3 cells showed higher numbers of cells in the G2/G1 phase (58.93% and 63.34%) compared to the non-targeting shRNA control (51.46%). These increases were coupled with a decreased percentage of cells in the G2/M phase. The sub-G1 population, which represents apoptotic cells, was not obviously observed in either L1CAM-proficient or -deficient cells. These data suggest that knockdown L1CAM expression led to decreased prostate cancer cell proliferation by inducing G0/G1 arrest.

### Overexpression of L1CAM in L1CAM-null prostate cancer cells promotes homotypic cell-cell adhesion and tumorigenicity

**Figure 6:**
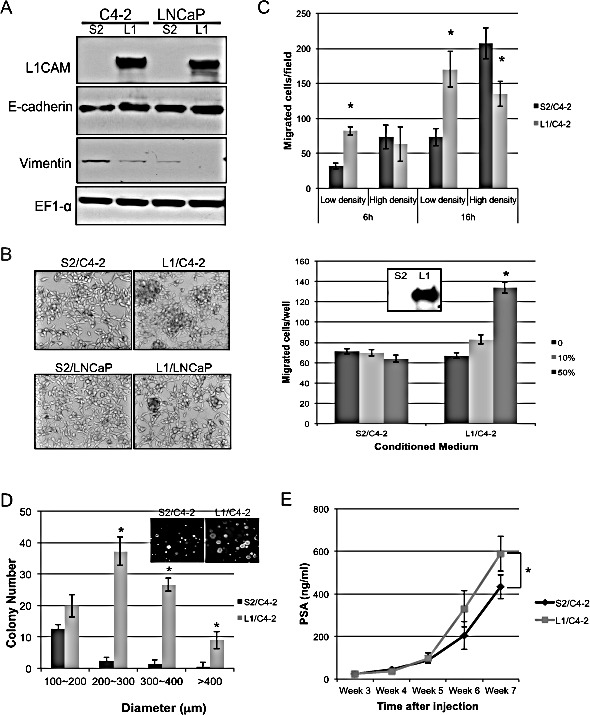
Effects of L1 cell adhesion molecule (L1CAM) overexpression in L1CAM-null prostate cancer cells (A) Western blot analysis of L1CAM and EMT-related markers E-cadherin and vimentin expression in C4-2 and LNCaP cells stably expressing L1CAM cDNA construct (L1) or empty vector control (S2). EF1-α protein levels are shown for various loading quantities. (B) Representative phase-contrast photographs (10x magnification) of the indicated cell lines in culture were taken 3 days after plating cells in single-cell suspensions. (C) Cell mobility of L1/ C4-2 and S2/C4-2 cell lines with low (2×10^4^ cells/well) or high (2×10^5^ cells/well) cell density at the indicated time points (top), and parental C4-2 cells (2×10^5^ cells/well for 16 hours) toward the indicated conditioned medium of transwells (bottom) was measured. Western blot analysis of L1CAM expression in the conditioned medium is shown in the box *p<0.05 compared with vector control group. (D) Colony formation of L1/C4-2 and S2/C4-2 cells was measured and compared by soft agar assay. Total number of colonies in each of the four size classes was counted, and results are expressed as the mean±SD of tripicate wells. * p<0.001, compared to the vector control group. A representative photograph (20x magnification) is shown in the box. (E) Tumorigenicity of L1/C4-2 cells *in vivo*. 5×10^5^ L1/C4-2 cells or S2/C4-2 were injected into the tibia of nude mice (n=6). Tumor growth was monitored by serum PSA weekly. *p<0.05 compared with the vector control group.

In order to study the biological function of L1CAM in a comprehensive manner, we took a genetic approach to overexpress L1CAM in LNCaP and its androgen-independent subline C4-2 that do not normally express L1CAM (Fig. 6A), and determined their behavior changes compared with the vector control. We found that L1CAM-overexpressing cells tend to form cell clumps in culture which caused uneven cell distribution after replating (Fig. 6B). This effect on cell-cell adhesion was accompanies by increased expression of E-cadherin and decreased expression of vimentin (Fig. 6A).

We examined whether the abundant L1CAM overexpression influences the mobility of prostate cancer cells by transwell migration assay. Interestingly L1/C4-2 moved toward the bottom chamber much slower than the vector control S2/C4-2 cells in the using high density of cells but significantly reversed when lower numbers of cells was assayed in a shorter time period to prevent cell-cell interactions. Moreover, the ectodomain of L1CAM shed in the conditioned medium of L1/C4-2 showed chemotactic activity for parental C4-2 cells (Fig. 6C). This result suggests a biphase function of L1CAM in the motility of C4-2 cells upon the avidity of homotypic cell-cell interaction. The inhibition of *in vitro* migration shown in confluent growing C4-2 cells was due to the enhanced intercellular adhesion among cells.

To examine whether the L1CAM-mediated homotypic cell interaction alters the tumorigenicity of prostate cancer cells, the growth rate of L1CAM/C4-2 was determined by *in vitro* proliferation and anchorage-independent colony formation assay. Although the growth rate of L1/C4-2 cells was very similar to that of the S2/C4-2 vector control cells when they grew on plastic dishes (Fig. S2B), L1/C4-2 cells were able to form more colonies >200 μm in soft agar compared with the S2/C4-2 cells (Fig. 6D). In addition, L1/C4-2 tumor progress faster than S2/C4-2 when cells implanted into mice tibias, as assessed by serum prostate specific antigen (PSA) (Fig. 6E). These data suggests an increased survival/growth advantage of L1CAM-expressing cells in anchorage-independent conditions.

### Liposome-encapsulated L1CAM siRNA treatment suppresses the growth of PC3 tumors in mouse bone

**Figure 7:**
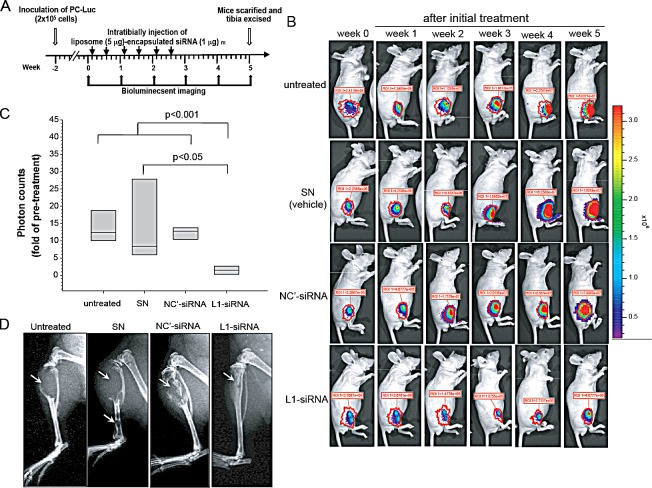
Therapeutic effects of L1 cell adhesion molecule (L1CAM)-siRNA in an experimental model of prostate cancer bone metastasis (A) Experimental scheme. As described in Materials and Methods. (B) Bioluminescent images of representative individual mice 1 day before treatment (week 0) and during the 5-week monitoring period after initial treatment. Signals were adjusted to the same color scale for the entire time course. Quantification of photon counts in the region of interest (ROI) of the legs is indicated. (C) The tumor volume at the end of the experiment was compared to that 1 day before treatment and presented as the multiples of change of photon counts of each individual. The plots display median values with the 25th and 75th percentiles (n=8). (D) Radiographs of representative mouse tibia in each group at the end of the experiment. PC3-Luc tumor-induced osteolytic lesions are indicated.

We further examined whether siRNA-mediated inhibition of L1CAM expression represents a promising antigrowth and antimetastatic strategy for prostate cancer gene therapy, particularly for bone metastases. PC3-Luc cells were inoculated into the tibia of nude mice and allowed to form tumors for 2 weeks. Once tumors had developed, as demonstrated by BLI, tumor-bearing mice received liposome SN-encapsulated L1CAM siRNA, negative control siRNA, or the SN vehicle alone intratumorally twice a week for 3 weeks, or else received no treatment (Fig. 7A). The responsiveness of PC3-Luc tumors to this therapy was monitored by BLI weekly (Fig. 7B). At the same time, mice were also assessed for any cytotoxic effects of siRNA delivery by recording body weights. Mice did not lose any significant body weight during the treatment period. A quantitative analysis of light emission revealed a 15-fold increase in the average photon counts of untreated and control groups at bony sites after the 5-week monitoring period, indicating aggressive tumor growth in control animals. Normalized signal progression levels (tumor growth) increased to a similar extent in the untreated group and both SN vesicle alone and control siRNA treatments, suggesting no therapeutic inhibition by these agents. In contrast, L1CAM siRNA therapy maintained a relatively constant or slowly changing profile of bioluminescence signals over time (Fig. 7C), with 2 of 8 (25%) of the tumors having no signal at 21 days after the first dosage. The reduction in PC3-Luc tumor growth by L1CAM siRNA was also confirmed by radiography (Fig. 7D), which indicated that PC3-Luc tumor-induced osteolytic lesions with destruction of the cortex had dramatically decreased in L1CAM siRNA-treated animals.

**Figure 8:**
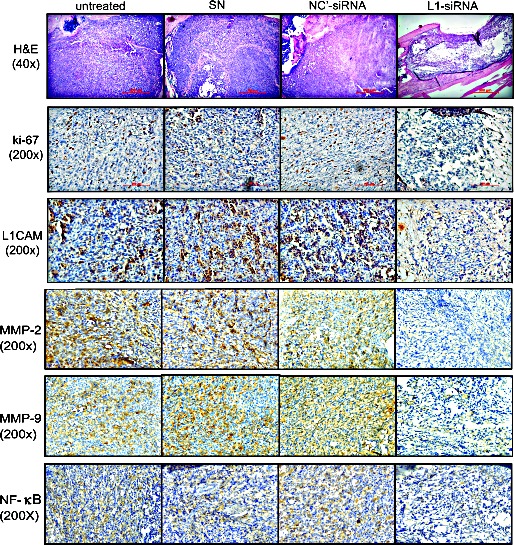
Histopathologic examination of PC3 bone tumors Mice were killed on day 50 after tumor cell implantation, and tumor-bearing legs were harvested and evaluated. Representative photomicrographs of the histopathological analysis (H&E; 40x) and immunohistochemical staining showing marked tumor regression and reductions in cell proliferation (ki-67) and protein expression of L1CAM, MMP-2, MMP-9 and NF-κb in bone specimens from animals receiving L1CAM-siRNA treatment (200x; scale bar=100 μm).

To better characterize the therapeutic effect of liposome-delivered L1CAM-siRNA, we excised the affected legs from all mice after 6 weeks of treatment and assessed pathologic changes in the tumors. A histological analysis (Fig. 8, H&E) revealed healthy and packed tumor cells growing in the marrow cavity of the control groups, either no-treatment or treated with SN vesicle alone or non-targeting siRNAs. Highly proliferative tumor cells in these control groups were confirmed by ki-67 expression (Fig. 8, ki-67). In contrast, extensive necrotic regions were found in tumors excised from L1CAM-siRNA treated animals, where few ki-67-stained cancer cells were detected. In addition, IHC staining of L1CAM showed extensive reduction of L1CAM expression in tumors treated with L1CAM siRNAs, indicating effective gene knockout in tumors by repeated intratumoral administration of liposome-encapsulated siRNA (Fig. 8, L1CAM). This inhibitory effect of administrated siRNA on L1CAM expression was strictly associated to decreased IHC staining of MMP-2, MMP-9 and NF-κb in cancer cells. Taken together, these results confirmed massive tumor regression and NF-κb pathway inhibition by L1CAM-siRNA therapy.

## DISCUSSION

Bone metastasis and skeletal complications are the major contributing factors to prostate cancer morbidity and mortality. Early detection of bone metastasis can help determine the best treatment strategy and prevent complications. Standard diagnostic algorithms of bone metastases primarily rely on ^99M^Tc methylene diphosphonate bone scintigraphy. The main problem with a bone scan is its low specificity, and its diagnostic effectiveness has been widely questioned in the literature [33, 34]. Indeed, in many cases, regions of increased uptake cannot definitively be characterized as negative or positive for malignancy. Although the prostate-specific antigen (PSA) test is widely used to clinically screen men for prostate cancer, and is known to be a sensitive marker for monitoring cancer progression, it also provides only limited information on the presence of bone metastasis in patients [35]. This study has, for the first time, shown that patients who develop skeletal metastasis from prostate cancer tend to have the highest levels of the L1CAM, a finding potentially useful for clinical practice in the early diagnosis of prostate cancer bone metastasis. The soluble form of the L1CAM was reported to be an active factor in angiogenesis, anti-apoptosis, and cell migration in various cancers [36-38]. In addition, the L1CAM was implicated in the chemoresistance of malignant tumor cells, including pancreatic ductal adenocarcinomas [39], glioblastoma multiforme [40], ovarian carcinoma [37], and anaplastic thyroid carcinoma [41]. One of the possible future applications of the L1CAM serum assay is its use as a prognostic tool before or after androgen deprivation therapy or radiation therapy for advanced disease progression. We anticipate that additional prospective follow-up studies using larger patient populations will establish the significant clinical utility of the serum L1CAM analysis.

Numerous studies have demonstrated the distinction of L1CAM's presence in cancerous *vs.* normal tissues in various cancer types, which is also associated with cancer progression [42]. In the present study, we found that unlike other cancer types, the L1CAM was barely expressed in localized prostate cancer, with no significant difference with that of normal glands. Notably, the localization of L1CAM protein was detected mostly at tumor-adjacent stroma but not in the central area of the tumor. Nevertheless, the L1CAM is thought to play key roles in prostate cancer metastasis as we demonstrated that its expression was strongly related to the metastatic potency of human prostate cancer cells, and its downregulation in cells resulted in insufficient cell migration, invasion, and colony formation *in vitro* and metastasis *in vivo*. Currently, the EMT is a well-accepted mechanism by which the L1CAM leads to a more-aggressive tumor phenotype that was demonstrated in a variety of cancer types [42]. In our study, L1CAM-mediated metastasis by prostate cancer cells was contradictory to EMT induction, and was even more likely associated with the mesenchymal-to-epithelial transition (MET), based on the expression patterns of E-cadherin and vimentin between L1CAM-deficient and L1CAM-proficient PC3 cells, and L1CAM-null and L1CAM-overexpressing C4-2 cells. Interestingly, L1/C4-2 cells exhibit low motility when cells grew in high but not low density to prevent cell-cell contact, whereas ectodomain of L1CAM secreted by L1/C4-2 in the conditioned medium promoted parental C4-2 cell migration in a dose-dependent manner. This result in conjunction with the data from L1CAM expression in clinical prostate cancer tissue array and patient sera suggests that in primary lesions of prostate cancer, the L1CAM may be characterized as a tumor suppressor due to its strong cell-adhesion function and in a protective role against the carcinoma-related EMT thereby tumor-associated stroma but not localized tumor cells may express and release L1CAM, a chemoattractant and adhesive matrix for cancer metastasis initiation. When tumor cells enter the circulation, the L1CAM expressed on the cell surface may allow cancer cells to aggregate and survive in blood stream and growth at distant metastatic sites through cell-cell and/or cell-ECM contact. Indeed, other than the homotypic cell adhesion shown in the present study, L1CAM can also mediate heterotypic interactions with activated endothelium and platelets by binding to multiple vascular and platelet integrins [43, 44], which implies on promoting cancer cell extravasation. Although the mechanism by which the L1CAM regulates EMT-related gene expression and confers enhanced motility and metastatic abilities to prostate cancer remains to be determined, our study provides a preliminary association between L1CAM expression and activation of the AKT/NF-κB signaling pathway, that may trigger cell growth and expressions of certain pro-metastatic genes, such as MMP-2 and MMP-9 as shown in this study. Indeed, resent literatures have revealed the critical role of NF-κB on the induction and maintenance of EMT via transcription regulation of mesenchymal genes encoding vimentin and the metalloproteinases MMP-2 and MMP-9, resulting in enhanced ability for cell migration and invasion. Moreover, AKT activation also contributes to the malignant phenotype of cancer cells in various ways [45], including stabilizing the cell cycle inhibitors p21Cip1 and p27Kip1 and inducing the translation of mRNAs for cyclins D1 and D3 [46, 47] to enhance cell cycle progression, overexpression of anti-apoptotic factors, such as Bcl-2, Bcl-xL and survivin [48] to promotes cell survival, and up-activation of NF-κB and regulation of MMP-2 and 9 activities [49, 50] to facilitate tumor cell invasion. The repression in a diverse AKT-downstream oncotargets by L1CAM-shRNA may override its effect on the expression of E-cadherin and vimentin, and lead to therapeutic intervention. A similar result was also documented in a recent publication [51], where L1CAM-mediated colon cancer metastasis required NF-κB signaling but did not rely on induction of the EMT. Therefore, there is a considerable cancer type-dependent effect of the L1CAM on the EMT-like phenotype. Future studies of gene array patterns induced by the L1CAM and AKT/NF-κB should provide more information on understanding the molecular mechanism of prostate cancer bone metastasis.

An antibody against the extracellular domain of the L1CAM was recently developed and was shown to inhibit the growth of L1CAM-expressing tumor cells including pancreatic and ovarian carcinoma and an intrahepatic cholangiocarcinoma in animal models [52-55]. However, subcellular localization of the L1CAM is not limited to the cell surface but is also reported in nuclei as a cleavage product containing only the intracellular domain [56]. The antibody-based transductional targeting approach described above, therefore, has the drawback of antagonizing the nuclear function of the L1CAM. In contrast, therapy of L1CAM-positive tumors using reagents that interfere with L1CAM-mediated intracellular signaling mechanisms may be more promising for improving classical therapeutic approaches. Molecular therapy using siRNA has shown great therapeutic potential for diseases caused by abnormal gene overexpression or mutations. We herein present a proof-of-principle study to illustrate that liposome-encapsulated L1CAM-siRNAs efficiently suppressed the growth of PC3 tumors in a bone xenograft model. These results strongly support our concept that the L1CAM is a potential therapeutic target in prostate cancer, and L1CAM-siRNA is a promising gene therapeutic approach for treating bone metastatic prostate cancers. SN liposome-mediated siRNA delivery with intratumoral administration demonstrated in the current model might not be clinically practical for skeletal metastases. Recently, a modified SN liposome, 1,2-dioleoyl-sn-glycero-3-phosphatidylcholine (DOPC), that was proven to have high efficacy in therapeutic siRNA delivery through an intraperitoneal or intravenous injection in human ovarian cancer xenograft models [57, 58], was formulated with an siRNA to target Eph2 gene expression for a phase I dose-escalation trial in patients with advanced cancer (http://www.clinicaltrials.gov/ct2/show/NCT01591356). Moreover, numerous clinical studies of liposome/lipoplex-based siRNA cancer therapeutics also provide promising data with no signs of nonspecific toxicity [59-61]. These clinical results combined with our present data have important implications for future translational applications of L1CAM-siRNA therapy to systemic cancer treatment.

## METHODS

### Cell cultures

Cell culture media and reagents were purchased from Invitrogen (Carlsbad, CA) unless otherwise specified. The human prostate cancer cell lines, LNCaP, C4-2, PC3, and DU145, were grown in T medium supplemented with 5% fetal bovine serum (FBS). Human prostatic epithelial PrEC cells were purchased from Lonza (Rockland, ME) and maintained in PrEGM™ (Lonza) according to the manufacturer's instructions. The HEK 293FT cell line that was used for producing recombinant lentiviruses was purchased from Invitrogen and grown in Dulbecco's modified Eagle medium (DMEM) supplemented with 10% FBS. Cells were cultured in a 37 °C incubator filled with 5% CO2 and were routinely passaged at 90% confluence.

### Real-time quantitative reverse-transcription polymerase chain reaction (RT-PCR)

Cellular RNA extraction and reverse-transcription were performed as previously described [62]. The real-time PCR was performed using a LightCycler 480 TaqMan master kit with gene-specific primers and the corresponding Universal Probe Library probe (Roche Applied Science, Mannheim, Germany). Sequences of primers and probes are shown in Supplemental Table S1. The conditions of the PCR were described in a previous study [16], and HSPCB was used as a housekeeping gene for normalizing the expression of each gene.

### Western blot analysis

Western blot analyses of cell lysates were performed as previously described [16] except that blots were probed with 1:1000-diluted primary antibodies for rabbit anti-MMP-2, MMP-9, anti-phospho-AKT and anti-AKT, anti-phospho-IKK, anti-IκBα, and anti- NF-κB p65 (Cell Signaling, Beverly, MA) or mouse anti-human L1CAM (NeoMarker, Fremont, CA), anti-E-cadherin (Cell Signaling), anti-vimentin (Santa Cruz Biotechnology, Santa Cruz, CA), and anti-fibronectin (BD Biosciences, San Jose, CA). For a loading control, blots were probed with an anti-EF1-α monoclonal antibody (1:10,000; R&D Systems, Minneapolis, MN). After incubation with horseradish peroxidase (HRP)-conjugated secondary antibody (1:5000, GE Healthcare Life Sciences), signals were detected by an enhanced chemiluminescence (ECL) Plus system (GE Healthcare, Pittsburgh, PA). Protein bands were quantified using ImageJ software.

### Immunohistochemical (IHC) staining

A prostate adenocarcinoma tissue microarray was obtained from IMGENEX (IMT-01254, San Diego, CA) and contained 95 tissue cores representing samples from 48 cases of prostate cancer and 15 matched normal adjacent tissues. Mice bone xenograft tissues were collected at the end of the animal experiments (5 weeks after initial treatment). IHC staining was performed using the Novolink Polymer Detection System (Leica Microsystems, Newcastle Upon Tyne, UK) as previously described [16]. Antibodies used in the study included mouse anti-human L1CAM (1:40; clone UJ127, NeoMarker), mouse anti-human ki-67 (1:50; NCL-Ki67-MM1, Leica Biosystems), mouse anti-MMP-2 (1:50; clone MMP2/8B4, LifeSpan Biosciences), rabbit anti-MMP-9 (1:100; AB19016, Millipore), and rabbit anti-human NF-κb (1:100; clone E379, Millipore). Scoring of immunostaining of L1CAM in tissue microarray was done by two pathologists from the Department of Pathology, Emory University School of Medicine (Atlanta, GA) using a semi-quantitative scoring method according to the staining intensity and area extent, which has been widely accepted and used in our previous studies [63, 64]. L1CAM expression was identified as any identifiable cytoplasmic/membranous staining, and quantified in 25% increments by visual estimation. Intensity of staining was recorded as weak, moderate and strong.

### Serum analysis

Human serum samples from prostate cancer patients with or without bone-scan-confirmed metastasis and a normal population control group, whose prostate biopsy showed no evidence of cancer and prostate-specific antigen levels were <4.0 ng/mL, were obtained from the Department of Urology, Emory University with informed consent under institutional review board-approved protocols. To detect soluble L1CAM, 96-well microtiter plates were coated with 2 μg/ml of a human L1CAM antibody (clone 5G3, BD Biosciences) overnight. Wells were blocked with 2% bovine serum albumin (BSA) for 2 h and then incubated for 2 h with serum (1:15 dilution). After five washes with 0.1% Tween 20, wells were incubated for 2 h with 1 μg/ml of a biotin-conjugated L1CAM antibody (clone UJ127, NeoMarker) followed by streptavidin-conjugated peroxidase (Sigma-Aldrich, St. Louis, MO) using 3,3′,5,5′-tetramethylbenzidine (TMB) as a substrate. The color reaction was stopped by the addition of 1 N HCl and analyzed at 450 nm using an enzyme-linked immunosorbent assay (ELISA) reader. Recombinant human NCAM-L1/Fc Chimera (R&D System) served as an internal standard for the assay.

### Flow cytometry

For surface L1CAM detection, cultured cells were harvested with Accutase Cell Detachment Medium (eBioscience, San Diego, CA) and then subjected to cell-surface marker staining as previously described [65], except that cells were incubated with 1 μg/ml of a mouse anti-human L1CAM antibody (clone 5G3) or mouse IgG2 isotype control antibody (BD Biosciences). For the cell-cycle analysis, cells were harvested with 0.25% trypsin and 0.02% EDTA, fixed with 100% cold ethanol, and then stained with 100 μg/mL propidium iodide in PBS containing 20 μg/mL RNAse (Sigma-Aldrich). The L1CAM cell-surface expression and cell-cycle distribution were analyzed using a FACSCalibur® Flow Cytometry System (Becton Dickinson, Franklin Lakes, NJ) with CellQuestPro software and the ModFit LT program, respectively.

### Cell-migration and -invasion assays

The invasion and motility of cancer cells were assessed using 24-well transwell plates. Briefly, 2×10^5^ cells in 0.5% FBS-containing media were added to the upper chamber with 8-μm pore polycarbonate (migration assay) or coated with 1 mg/ml of Matrigel (invasion assay), and the lower chamber was filled with growth medium. After incubation for 16 h, the upper surfaces of the membranes were scrubbed with a cotton-tipped swab. Invading and migrating cells on the lower surfaces of the membranes were fixed and stained with 0.5% crystal violet. Random fields (10/membrane) were photographed at 20x magnification and quantified by cell counting.

### RNA interference and L1CAM gene overexpression

For small interfering RNA (siRNA) transfection, the siRNA sequence targeting coding region 298~883 of human L1CAM (L1-siRNA) was chosen and confirmed to be a good target using the software developed by Ambion. A mammalian non-targeting siRNA control (NC'-siRNA) was also designed using enhanced green fluorescent protein (EGFP) sequences. The siRNAs were prepared using *in vitro* transcription and a Dicer kit (Invitrogen) according to the manufacturer's instructions. siRNAs were transfected into PC3 cells with the lipofectamine 2000 reagent (Invitrogen). To generate a stable L1CAM gene knockdown PC3 cell line, a short hairpin (sh)RNA lentiviral expression system was used. RNA interference vectors, pLKO.1-shL1CAM (clone B, TRCN0000063914, target sequence, GCCAATGCCTACATCTACGTT; clone E, TRCN0000063917, target sequence GCTAACCTGAAGGTTAAAGAT) and control pLKO.1-shGFP (TRCN0000072178, target sequence: 5′-CAACAGCCACAACGTCTATAT-3′) were obtained from the National RNAi Core Facility (Institute of Molecular Biology, Academia Sinica, Taipei, Taiwan). Recombinant lentiviruses carrying shRNA were produced as previously described [16]. PC3 cells were infected with recombinant lentiviruses, and stable cell lines were selected with 2.5 μg/ml puromycin for 1 week. To establish L1CAM-expressing LNCaP and C4-2 cells, L1CAM cDNA was amplified from PC3 cell RNA and then cloned into the S2 bicistronic retroviral vector [66], in which L1CAM was driven by a retroviral long terminal repeat promoter. Retroviral production and infection were performed as described previously [65]. The resultant stable cell lines were selected with 0.8 mg/ml G418 (Calbiochem, La Jolla, CA, USA) for 10 days.

### Gelatin zymography

MMP-2 and MMP-9 enzymatic activity was determined using a 10% gelatin zymography sodium dodecylsulfate polyacrylamide gel electrophoresis (SDS-PAGE) system (Invitrogen) according to the manufacturer's instruction. Briefly, conditioned medium was mixed with 2x nonreducing sample buffer and separated on a 10% polyacrylamide gel containing 0.1% gelatin. After electrophoresis, the gel was washed and incubated in Zymogram Renaturing Buffer for 30 min at room temperature with gentle agitation to renature the gelatinases. After washing twice, the gel was incubated for 18 h in Zymogram Developing Buffer, and subsequently stained by SimplyBlue™ SafeStain. Blue-stained bands were visible on a clear gel after destaining with water. An image of the gel was detected with the Gel Doc XR system (Bio-Rad Laboratories, Hercules, CA).

### Cell-aggregation assay

Cells were grown to 70% confluence and then harvested with Accutase Cell Detachment Medium. Resuspended cells at 5×10^5^ were added to 60-mm polyHEMA-coated Petri dishes and then agitated at 80 rpm for different periods of time. Resultant cells were subsequently examined under an inverted phase-contrast microscope and photographed in five different fields. The degree of aggregation was determined by counting clusters consisting of more than 10 cells.

Colony-formation assay. Parental and shRNA stably expressing PC3 cells (100 cells) were seeded in duplicate in 6-well plates. The medium was replaced with fresh medium every 3 days. After being cultured for 10 days, cells were fixed with 4% paraformaldehyde for 15 min, washed with phosphate-buffered saline (PBS), stained with 0.1% crystal violet for 15 min and photographed. Each experiment was independently performed three times.

### Anchorage-independent growth assay

The anchorage-independent growth of L1/C4-2 and S2/C4-2 were examined by clonogenic cell growth on soft agar. Briefly, each well of a 6-well plate was first layered with 0.6% agarose in growth medium (DMEM supplement with 10% FCS). The cell lines to be tested were trypsinized, and 10^4^ cells were resuspended in growth medium containing 0.3% agarose and then were poured as a top layer in the 6-well plates. The plates were incubated at 37°C for 10 days. Colonies were counted and the diameter of colonies was scored under microscope using Openlab 4.03 software (Improvision, MA).

### Luciferase assay

PC3 cells were cotransfected with a 3x NF-κB promoter luciferase reporter plasmid and pCMV-βgal (galactosidase) in a 5:1 molar ratio using the lipofectamine 2000 transfection reagent. After 48 h of incubation, cell extracts were prepared for the luciferase and β-gal activity assessments respectively using the Luciferase Assay System and β-Galactosidase Enzyme Assay System (Promega, Madison, WI) according to the manufacturer's instructions. The relative luciferase activity was calculated by dividing the firefly luciferase relative light units (RLU) by the corresponding value for β-gal activity present in each sample. Three independent experiments were performed in triplicate.

### Animal studies

All animal work was done in accordance with a protocol approved by the Institutional Animal Care and Use Committee of China Medical University (Taichung, Taiwan). Six-week-old male athymic nude mice (BALB/cAnN.Cg-Foxn1nu/CrlNarl) were obtained from the National Laboratory Animal Center (Taipei, Taiwan). To evaluate the effect of the L1CAM on prostate cancer metastasis, 10^5^ PC3-Luc cells that were transfected with L1-siRNA, NC'-siRNA, or lipofectamine 2000 only (mock) for 24 h were suspended in 100 μl of sterile PBS and injected into the left ventricle of anesthetized nude mice using a 1-mL syringe with a 25G needle. A successful intracardiac injection was immediately confirmed after tumor cell injection with the IVIS Imaging System (Caliper Life Sciences, Hopkinton, MA, USA) as described previously [65] if images showed systemic bioluminescence distributed throughout the animal. Only mice with evidence of a satisfactory injection were used in the experiment. Subsequent metastasis was assessed and monitored once a week by bioluminescent imaging (BLI) from both dorsal and ventral views for up to 7 weeks. To evaluate the efficacy of siRNA therapy, 2×10^5^ PC3-Luc cells in a final volume of 10 μl of PBS were injected into the mouse tibia at a tilted angle of 45°. Two weeks after cell injection, PC3-Luc tumor-bearing mice were randomized and received liposome SN-encapsulated L1CAM-siRNA, NC'-siRNA, or SN alone (vehicle control) intratumorally twice per week for 3 weeks or else no treatment (n=8). Tumor growth was monitored by BLI weekly. Animals were sacrificed at 5 weeks after the initial treatment. Before being sacrificed, radiographic images were taken with a specimen radiography system (Faxitron X-ray, Wheeling, IL).

### Statistical analysis

All data are presented as the mean±standard deviation (SD) unless otherwise specified. Differences between groups were analyzed using the two-tailed Student's t-test or one-way analysis of variance (ANOVA) for multiple comparisons. A p value of <0.05 was defined as statistically significant.

## SUPPLEMENTARY MATERIAL TABLE, FIGURES


