# Supplementary material for: Targeting L1 cell adhesion molecule expression using liposome-encapsulated siRNA suppresses prostate cancer bone metastasis and growth
Source: Oncotarget. 2014 Sep 16;5(20):9911–29. doi: 10.18632/oncotarget.2478 (PMC4259447; doi:10.18632/oncotarget.2478)
Supplement: Supplementary file 1 [file oncotarget-05-9911-s001.pdf]

Targeting L1 cell adhesion molecule expression using liposome-encapsulated siRNA suppresses prostate cancer bone metastasis and growth

Supplementary Material

Table S1: List of primers and probes used in quantitative PCR

Gene	Primer sequences*	UPL[†]
L1CAM	F: ACCTTCGGCGAGTACAGGT<br>R: TTGATGTCCCCGTTGAGC	No.66
MMP-2	F: ATAACCTGGATGCCGTCGT<br>R: AGGCACCCTTGAAGAAGTAGC	No.70
MMP-9	F: GAACCAATCTCACCGACAGG<br>R: GCCACCCGACTGTAACCATA	No.53
E-cadherin	F: CCCGGGACAACGTTTATTAC<br>R: GCTGGCTCAAGTCAAAGTCC	No.35
Vimentin	F: AAAGTGTGGCTGCCAAGAAC<br>R: AGCCTCAGAGAGGTCAGCAA	No.16
Snail	F: GCTGCAGGACTCTAATCCAGA<br>R: ATCTCCGGAGGTGGGATG	No.11
Slug	F: TGGTTGCTTCAAGGACACAT<br>R: GTTGCAGTGAGGGCAAGAA	No.7
HSPCB	F: AGCCTACGTTGCACTATTACG<br>R: GAAAGGCAAAAGTCTCCACCT	No.55

* F: forward primer; R: reverse primer
[†] Universal Probe Library (Roche Applied Science)

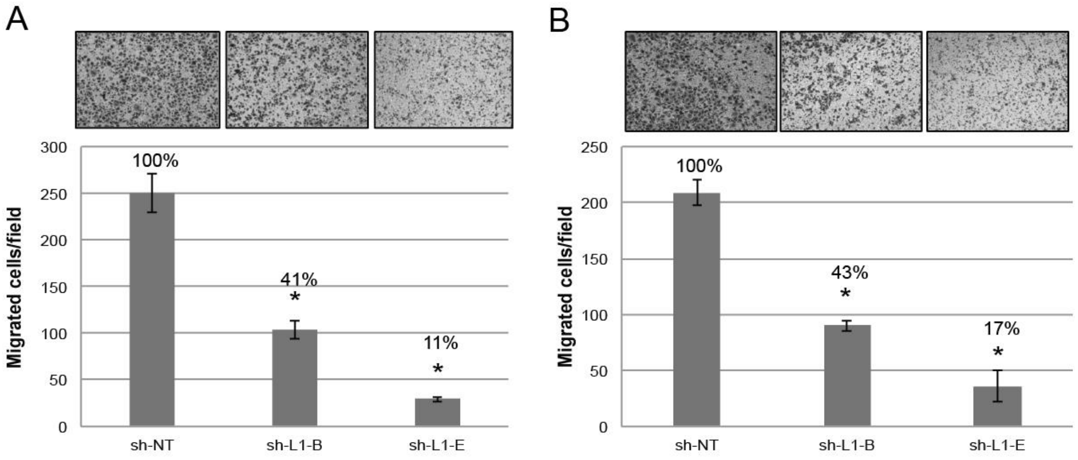

Supplementary Fig. S1: Comparison of cell migration of PC3 cells stably expressing shRNA constructs targeting L1CAM (L1-B and L1-E) or a non-target control (NT) under different culture condition of transwell migration assay. (A) $2x10^5$ cells in 0.5% FBS-containing media were added to the upper chamber and the migrated cell were counted 8 h after incubation. (B) $5x10^4$ cells in 0.5% FBS-containing media were added to the upper chamber and the migrated cell were counted 16 h after incubation. Assays were performed in three independent experiments in triplicates. Data are presented as the mean±SD of one representative experiment, and representative images (200x) of each line are shown at the top.

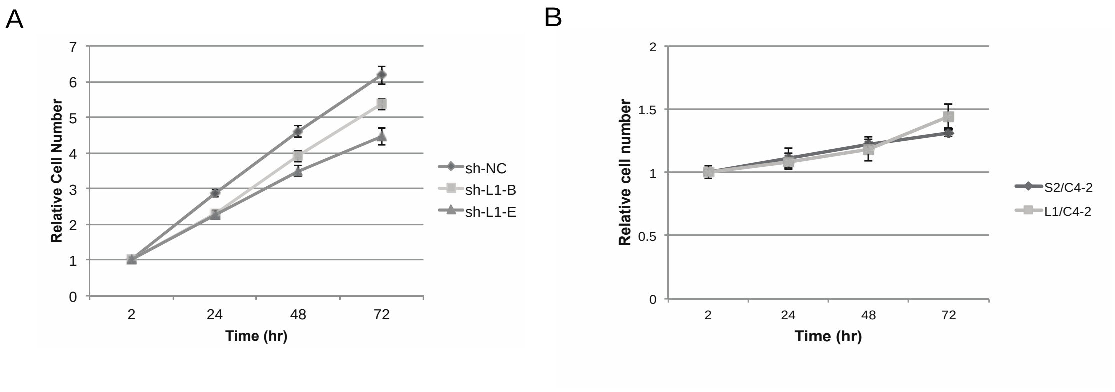

Supplementary Figure S2: Comparison of cell proliferation between L1CAM gene-modulated prostate cancer cells and corresponding control cells of (A) PC3 derivatives and (B) C4-2 derivatives by WST-1 assay performed daily for 3 days. The relative cell number was assessed by absorbance at 450 nm and presented as the fold change relative to the day of plating (2 hr). Error bars indicate SD of triplicate measurements.

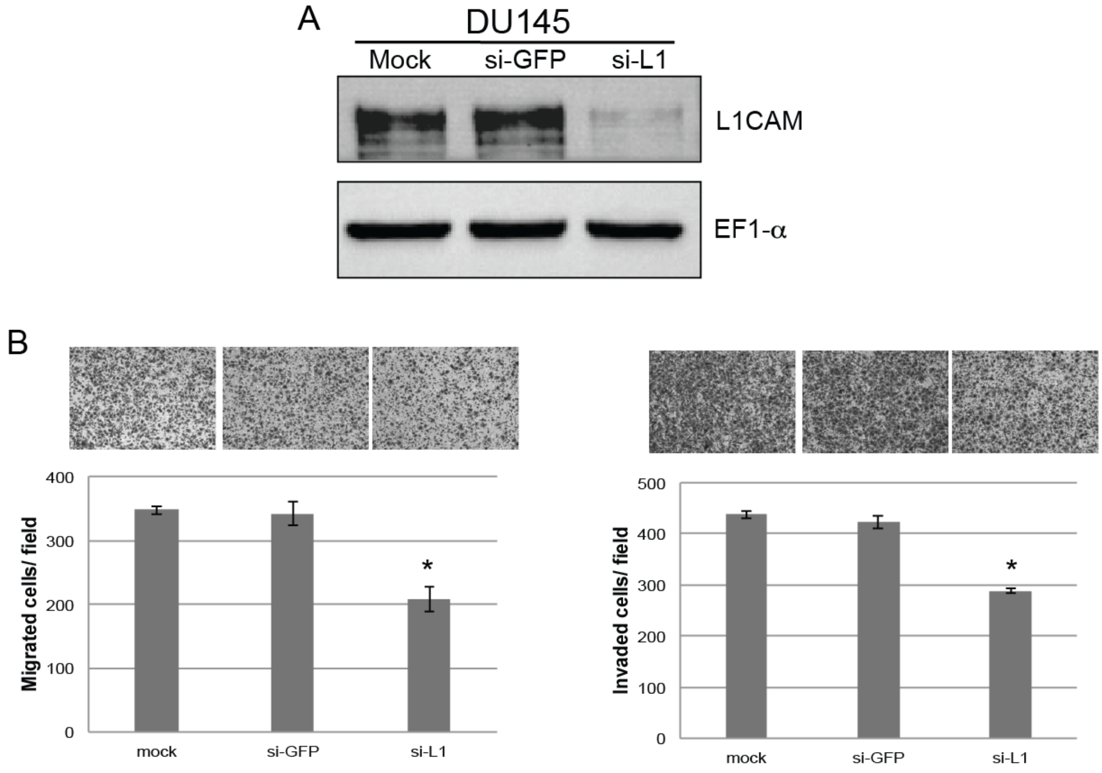

Supplementary Figure S3: Effects of L1 cell adhesion molecule (L1CAM) gene knockdown on the migratory and invasive abilities of DU145 prostate cancer cells. (A) Western blot analysis of L1CAM expression in DU145 cells transfected with L1CAM-siRNA (si-L1), EFGP negative control siRNA (si-GFP), or vesicle alone (mock). EF1-α protein levels are shown for various loading quantities. (B) Migration and invasion assays of L1CAM-siRNA-transfected PC3 cells. Cells that had migrated through the membrane (migration, left) or Matrigel (invasion, right) were stained with crystal violet and counted 8 h after cell plating. Assays were performed in three independent experiments in triplicates. Data are presented as the mean±SD of one representative experiment, and representative images (200x) of each line are shown at the top; * $p \leq 0.001$.

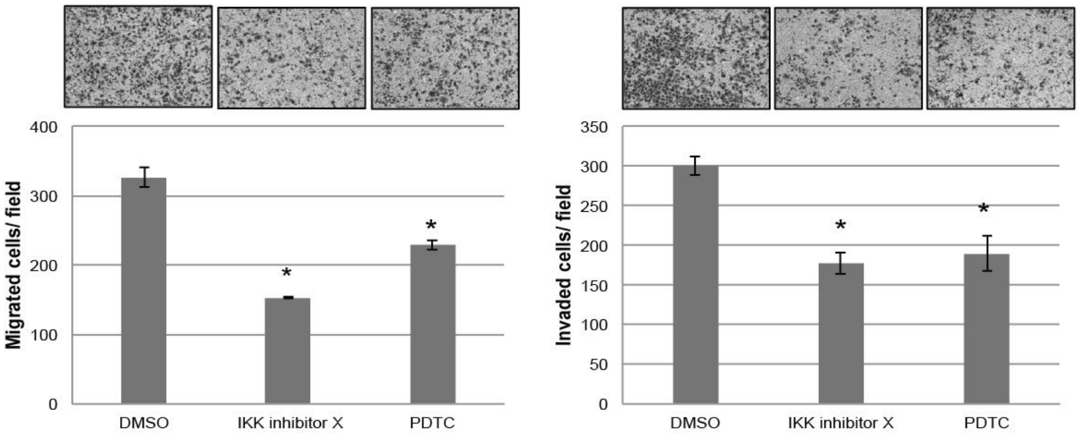

Supplementary Figure S4: Effects of signaling inhibitors on the migratory and invasive abilities of PC3 prostate cancer cells. $2x10^5$ serum-starved PC3 cells in the presence of 10 μM IKK inhibitor (IKK inhibitor X), NF-κB inhibitor (PDTC) or vehicle (DMSO) were added to the upper chamber of transwells. After incubation for 8 h, the migrating (left) and invading cells (right) on the lower surfaces of the membranes were stained with 0.5% crystal violet and counted. Assays were performed in three independent experiments in triplicates. Data are presented as the mean±SD of one representative experiment, and representative images (200x) of each condition are shown at the top; * $p \leq 0.001$.
